# Nuage in color: Systematic protein tagging shows the compositional complexity of germ granules

**DOI:** 10.1016/j.devcel.2025.03.015

**Published:** 2025-04-21

**Authors:** Laura L. Thomas, Devavrat M. Bodas, Geraldine Seydoux

**Affiliations:** HHMI and Department of Molecular Biology and Genetics, Johns Hopkins University School of Medicine, Baltimore, MD 21205, USA

## Abstract

In this issue of *Developmental Cell*, Huang et al. generate a library of *C. elegans* strains to systematically characterize germ granule composition. This survey catalogs condensate proteins in an intact organism using endogenous tags and sets the stage for future studies of condensate composition and function.

Germ granules are a class of biomolecular condensates found in germ cells. Electron microscopists in the 1970s described germ granules as dense, amorphous perinuclear assemblies and coined the term “nuage” (French for cloud)^1^. Since then, many proteins involved in small RNA biogenesis and mRNA regulation have been reported to concentrate in nuage. In the last decade, high-resolution microscopy studies in *C. elegans* revealed that nuage represents a collection of compositionally distinct condensates that adsorb to each other and the nuclear surface (Figure 1A)^2^. The exact number, organization, and composition of nuage condensates, however, has remained unclear. In this issue of *Developmental Cell*, Huang et al. present the most thorough analysis of nuage organization to date^3^.

This study represents a formidable undertaking to build and analyze a library of 80 strains representing 65 proteins. 59 were tagged at their endogenous loci using CRISPR genome engineering; this approach avoids transgenes that are often used in systematic surveys but can be prone to artefacts due to overexpression. The authors also checked the functionality of the tagged proteins by examining homozygous lines for fertility defects and, for some genes, by comparing different tags at the same locus. Finally, multicolor imaging and quantitative image analyses were used to compare the distribution of each tagged protein against a set of standards. In total, the survey assigned 52 proteins to 7 condensates (Figure 1), each defined by a unique composition and location within nuage. Huang et al. also used the library to begin to unravel the hierarchy of condensate interactions that drive nuage assembly. They report that loss of a critical component of P granules, the LOTUS domain protein MIP-1/EGGD-1, causes P granules and three associated condensates to re-localize to the cytoplasm (Figure 1A). In contrast, P-bodies, D granules, and E granules remain at the nuclear periphery, with D and E granules fusing. These observations suggest that condensates use different strategies to localize in nuage, with some condensates piggybacking on others to reach the nuclear periphery and some condensates relying on interactions within nuage to demix from each other.

What determines condensate composition? As expected, proteins that co-assemble in the same macromolecular complex were found to co-localize in the same granule (e.g., the PICS complex in the E granule). Proximity labeling experiments have been used to identify constituents of P granules, Z granules, and *Mutator* foci^4,5^. Cross-referencing these datasets with localization data from Huang et al. reveals that only a minority of nuage proteins interact primarily with proteins in their respective granule (Figure 1B). Most appear promiscuous, interacting with partners across several condensate types. Because nuage proteins also exist as soluble molecules in the cytoplasm, we do not know whether interactions revealed by proximity labeling occur in the condensate, in the cytoplasm, or both. Several theories are being developed to describe the rules that govern condensate composition^6^, the Huang et al. library provides a unique *in vivo*-verified dataset to test and refine these models.

What is the function of nuage? The multilayered organization of nucleoli has been proposed to facilitate the ordered processing and assembly of rRNA into ribosomes^7^. Similarly, the layered architecture of nuage is often presumed to facilitate regulation of nascent transcripts as they emerge from germ cell nuclei^2^. In this model, nascent transcripts exit nuclear pores directly into nuage condensates where they are scanned by Argonautes before sorting into other nuage compartments for translational licensing, sRNA (small RNA) amplication, or mRNA degradation. Consistent with this model, nuage condensates overlay clusters of nuclear pores and this association depends on FG (phenylalanine glycine) repeats in nucleoporins and the P granule protein GLH-1^8,9^Challenging this model is the observation that *mip-1*/*eggd-1* mutants remain fertile despite grossly disrupting nuage organization^8^. Two studies recently reported that mutants lacking the FG-repeat nucleoporin NPP-14 (human Nup214) disrupt nuage organization^8,9^. Like *mip-1*/*eggd-1* mutants—*npp-14*, and *npp-14*; *mip-1*/*eggd-1* double mutants are fertile and mis-regulate only a subset of transcripts, confirming that wild-type nuage architecture is not essential for the bulk of mRNA regulation. In contrast, mutants that dysregulate the activity of enzymes enriched in nuage exhibit both abnormal nuage and fertility defects. These observations suggest that biological activity drives nuage architecture, as has also been argued for the nucleolus^10^. One possibility is that high transcriptional activity in germ cells causes ribonucleoprotein (RNP) complexes to exceed their solubility limit in the cytoplasm, causing them to demix into condensates. Because RNP complexes share a subset of RNA and protein components, the condensates adsorb to each other and to the nuclear periphery where nascent RNA concentration is highest. This hypothesis does not exclude the possibility that condensate interactions within nuage feedback on biological activity to fine-tune enzymatic reactions and increase robustness. For example, the high concentration of nuclear pores under each nuage assembly could create a strong outward RNA flux that increases the efficiency of mRNA scanning and processing before release to the cytoplasm. A challenge for the future will be to disentangle effects caused by the loss of a specific condensate protein from effects caused by the loss of a condensate or a condensate-condensate contact.

Although much has been learned using *in vitro* approaches, the principles that govern the composition and function of condensates remain poorly understood. The Huang et al. library will be an invaluable resource for future cell biological and biochemical analyses of nuage. This study also benchmarks experimental standards (CRISPR tagging and quantitative multicolor colocalization analyses) for future surveys of condensates in systems beyond *C. elegans*. We can look forward to many more colorful rainbows lighting up nuage and other condensate assemblies in cells.

## Figures and Tables

**Figure 1.**
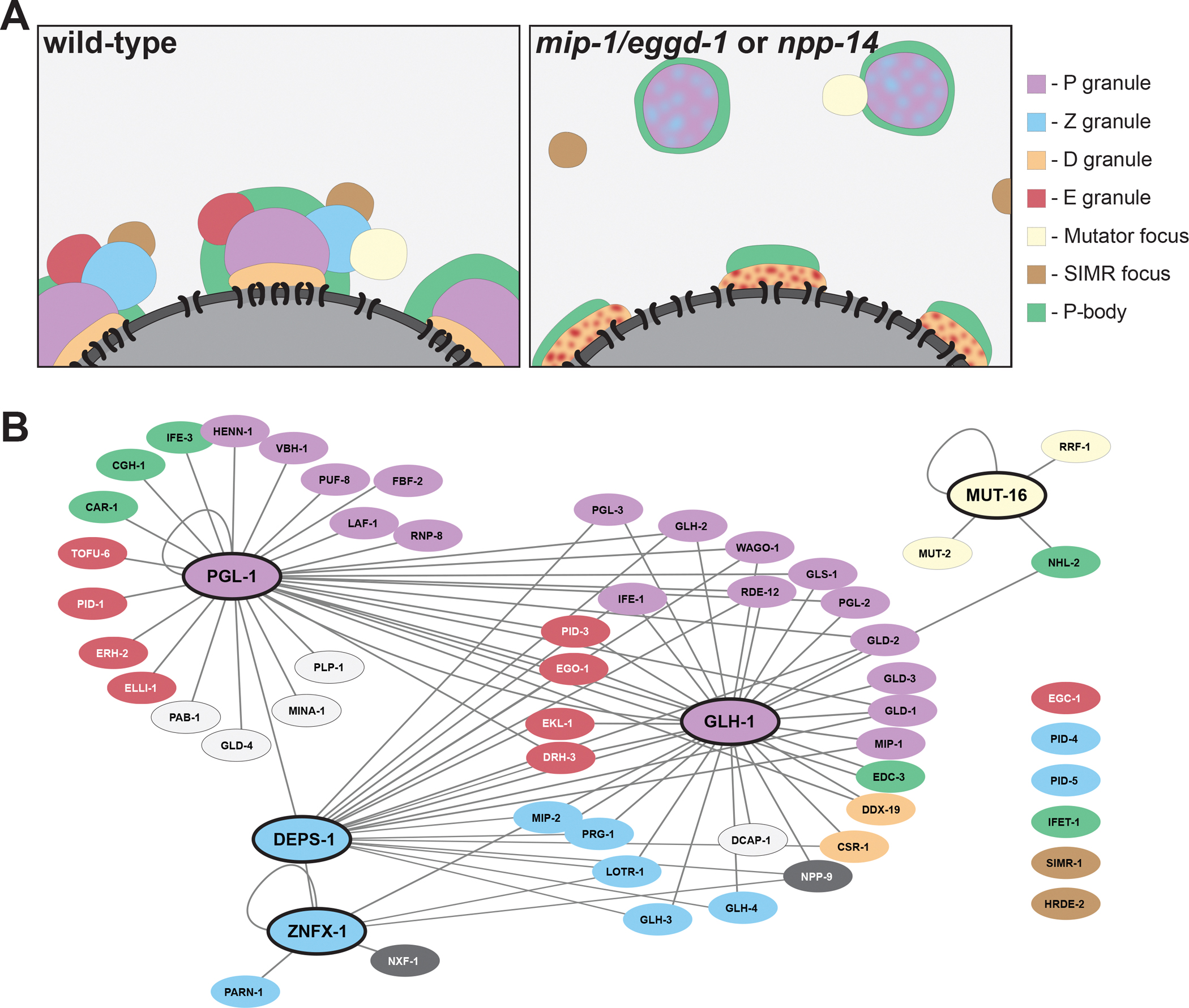
Architecture and interactome map of *C. elegans* nuage (A) Cartoon depicting nuage assemblies around a *C. elegans* germ cell nucleus (pachytene stage). Each assembly contains several condensate types that occupy a stereotypical position relative to each other, although exact positions vary and not all nuage assemblies contain all condensates. In *mip-1*/*eggd-1* and/or *npp-14* mutants, P granules, Z granules, *Mutator* foci, and SIMR foci detach from the nuclear periphery and nuclear pores no longer cluster. In those mutants, P and Z granules in the cytoplasm, and D and E granules on nuclei, mix with one another^8,10^. (B) Interaction network based on data generated by proximity labeling experiments using ZNFX-1, DEPS-1, GLH-1, PGL-1, or MUT-16 as baits^4,5^. Only proteins analyzed in the Huang et al. study are shown and each is colored based on its assigned condensate location. Proteins in white could not be assigned to a specific condensate. Six nuage proteins (lower right) were not recovered in the proximity labeling screens.

## References

[1] Voronina E, Seydoux G, Sassone-Corsi P, and Nagamori I (2011). RNA Granules in Germ Cells. Cold Spring Harbor Perspectives in Biology 3, a002774–a002774. 10.1101/cshperspect.a002774. 21768607 PMC3225947

[2] Phillips CM, and Updike DL (2022). Germ granules and gene regulation in the *Caenorhabditis elegans* germline. Genetics 220, iyab195. 10.1093/genetics/iyab195. 35239965 PMC8893257

[3] Huang X, Feng X, Yan Y-H, Xu D, Wang K, Zhu C, Dong M-Q, Huang X, Guang S, and Chen X (2024). Compartmentalized localization of perinuclear proteins within germ granules in C. elegans. Developmental Cell, S153458072400738X. 10.1016/j.devcel.2024.12.016. 39742661

[4] Price IF, Hertz HL, Pastore B, Wagner J, and Tang W (2021). Proximity labeling identifies LOTUS domain proteins that promote the formation of perinuclear germ granules in C. elegans. eLife 10, e72276. 10.7554/eLife.72276. 34730513 PMC8616582

[5] Zhao C, Cai S, Shi R, Li X, Deng B, Li R, Yang S, Huang J, Liang Y, Lu P, (2024). HERD-1 mediates multiphase condensate immiscibility to regulate small RNA-driven transgenerational epigenetic inheritance. Nat Cell Biol 26, 1958–1970. 10.1038/s41556-024-01514-8. 39354132

[6] Holehouse AS, and Alberti S (2025). Molecular determinants of condensate composition. Molecular Cell 85, 290–308. 10.1016/j.molcel.2024.12.021. 39824169 PMC11750178

[7] Lafontaine DLJ, Riback JA, Bascetin R, and Brangwynne CP (2021). The nucleolus as a multiphase liquid condensate. Nat Rev Mol Cell Biol 22, 165–182. 10.1038/s41580-020-0272-6. 32873929

[8] Thomas LL, Bodas DM, and Seydoux GC (2025). FG repeats drive co-clustering of nuclear pores and P granules in the *C. elegans* germline. Development, dev.204585. 10.1242/dev.204585. PMC12050070 40067309

[9] Lu P, Deng B, Li X, Niu X, Qiu Y, Liang Y, Liang Y, Tang G, Yuan Z, Luo G, (2025). A nuclear pore-anchored condensate enables germ granule organization and transgenerational epigenetic inheritance. Nat Struct Mol Biol. 10.1038/s41594-025-01515-7. 40082670

[10] Tartakoff A, DiMario P, Hurt E, McStay B, Panse VG, and Tollervey D (2022). The dual nature of the nucleolus. Genes Dev. 36, 765–769. 10.1101/gad.349748.122. 36342833 PMC9480854

